# BMI differences among in-hospital management and outcomes in patients with atrial fibrillation: findings from the Care for Cardiovascular Disease project in China

**DOI:** 10.1186/s12872-020-01544-8

**Published:** 2020-06-05

**Authors:** Fuxue Deng, Yan Zhang, Qiang Zhao, Yangyang Deng, Shanshan Gao, Lisha Zhang, Mengya Dong, Zuyi Yuan, Xinjun Lei, on behalf of the CCC-AF investigators

**Affiliations:** 1 Department of Cardiovascular Medicine, the Second Affiliated Hospital of Xi’an Jiaotong University, Xi’an, Shaanxi People’s Republic of China; 2 Department of Cardiovascular Medicine, the First Affiliated Hospital of Xi’an Jiaotong University, 277 Yanta West Road, Xi’an, Shaanxi 710061 People’s Republic of China; 3 Key Laboratory of Environment and Genes Related to Diseases, Xi’an Jiaotong University, Ministry of Education, Xi’an, Shaanxi People’s Republic of China; 4 Key Laboratory of Molecular Cardiology, Shaanxi Province, Xi’an, Shaanxi People’s Republic of China

**Keywords:** Atrial fibrillation, Body mass index, Medical care, Clinical outcomes

## Abstract

**Background:**

Underweight or obese status influences the prognosis of atrial fibrillation (AF). However, the association between stratification of body mass index (BMI) and in-hospital outcomes in patients with AF, remains lacking in China.

**Methods:**

Using data from the Improving Care for Cardiovascular Disease in China-AF project, which was launched in February 2015 and recruited 150 hospitals in China, we compared characteristics, in-hospital treatments and clinical outcomes among the stratifications of BMI for Asians.

**Results:**

A total of 15,867 AF patients with AF were enrolled, including 830 (5.23%) underweight, 4965 (31.29%) with normal weight, 3716 (23.42%) overweight, 5263 (33.17%) obese class I and 1093 (6.89%) obese class II participants. Compared with normal weight patients, underweight, overweight, and obese patients showed increased percentages of CHADS_2_ scores (3–6) and CHA_2_DS_2_-VASc scores (5–9). During hospitalization, overweight or obese patients showed greater use of rhythm control medications, anticoagulant drugs, and intervention therapies than underweight–normal weight patients. In adjusted logistic models, BMI was a strong predictor of in-hospital mortality. Especially, underweight BMI was associated with higher incidence of in-hospital mortality, with an adjusted odds ratio of 2.08 (95% confidence interval, 1.56–4.46; *p* = 0.04) than overweight and obese BMI.

**Conclusions:**

Asian patients with AF and high BMI received more medical treatments and presented less adverse in-hospital outcomes compared with those with underweight–normal weight. Although low BMI may be associated with other comorbidities and advanced age, underweight BMI retained a negative correlation with all-cause mortality in the patients with AF during hospitalization.

## What is new?

- This multicenter population-based cohort from 150 hospitals distributed throughout almost all regions of China is the largest contemporary registry study that explored BMI–related differences in clinical characteristics, management, and outcomes of patients with atrial fibrillation (AF).
- Patients with BMI > 23 in China required more frequent oral medical treatments and intervention strategies and presented lower in-hospital mortality rate than normal–underweight patients.

## What are the clinical implications?

- The quality of care for low–BMI patients hospitalized for AF in China should be improved using evidence-based treatments and strategies for prevention.
- Low BMI patients exhibit poorer in-hospital mortality compared with normal–weight or overweight patients. Thus, improving weight management may aid in reducing the observed weight differences in in-hospital mortality.
- The association between abdominal obesity and fat distribution with AF in China must be further investigated.

## Background

Atrial fibrillation (AF) is the most common sustained arrhythmia and resulted in high prevalence of stroke, all-cause mortality, and heart failure [1]. Both Asian populations and Western countries encounter increasing numbers of new-onset AF (NOAF), which is affected by several demographic and clinical risk factors, including age, male sex, obesity, and the presence of coronary artery disease (CAD) [2]. By 2050, the estimated number of patients with AF will reach 72 million, and almost 3 million may experience AF-associated stroke [3].

In China, limited studies have assessed the association between body weight/body mass index (BMI) and the quality of care and outcomes among patients with AF. Contemporary evidence in 2017 [4] from Korea proved that abdominal obesity is a potential modifiable risk factor for AF in non-obese Asian individuals. Similar results were observed in a population of young women from Denmark [5], suggesting that interventions to decrease abdominal obesity may reduce the population burden of AF. Furthermore, in Korean research, being underweight is associated with biological effects that contribute to AF development [6]. However, few studies have systematically compared the clinical characteristics and treatments of patients with AF according to BMI stratification in the current year. The relation of BMI and in-hospital quality of care to poor short-term outcomes in patients with AF in China remains unclear.

To avoid the limited representativeness of hospitals and shortage of quality control in previous studies launching the clinical practice of AF in China [7, 8], the Chinese Society of Cardiology (CSC) and the American Heart Association (AHA) initiated the Improving Care for Cardiovascular Disease in China (CCC)-AF project. The CCC-AF was developed on the basis of the guideline initiative of AHA [9]. Using data from this timely feedback database, we performed a population-based evaluation to investigate BMI differences in clinical characteristics, management during hospitalization and discharge, and in-hospital mortality among patients hospitalized for AF.

## Method

### Study population

Patients with established or newly diagnosed AF and who visited one of 150 tertiary hospitals from 30 provinces were recruited. Every tertiary hospital included in the CCC-AF project has at least 120 patients with a primary diagnosis of AF annually [10]. Exclusion criteria comprised patients with AF due to reversible conditions (e.g., untreated thyroid disease and pulmonary embolism) and with no record of BMI. In accordance with the exclusion criteria above, we analyzed data from 15,867 patients from February 2015 to March 2017 with an AF diagnosis (International Classification of Diseases, Ninth Revision [ICD-9]: 427.31; ICD, Tenth Revision [ICD-10]: I48.0%, I48.2%, I48.91%) before or upon admission. The CCC-AF project was registered at www.ClinicalTrials.gov (Trial registration number: NCT02309398) with the official title “Improving Care for Cardiovascular Disease in China: A Collaborative Project of AHA and CSC-Atrial Fibrillation”. It is an observational case-control and retrospective study that started in January 2015.

### Data collection

Baseline characteristics and clinical data were collected in accordance with the American College of Cardiology (ACC)/AHA recommendations [11] on data standards for clinical research on AF. Using Oracle Clinical Remote Data Capture system (Oracle Corporation, Redwood City, CA, USA) and a web-based platform, participating instructions were informed to submit consecutive eligible patients. Four third-party clinical approaches were used to monitor the accuracy and completeness of data collection. The supervision approaches consisted of face-to-face training workshops prior to data entry, a standardized online reporting tool, on-site quality control from a third party, and monthly inspections for data completeness as a monthly report. Details of the design, methodology, and quality control of CCC-AF project were published [10]. The objectives of the program include understanding of the current situation and main problems for the management of inpatients with AF, the assessment of the performance of the current quality improvement strategy, and the exploration and refinement of an optimal approach to promote clinical management. As a collaborative project of the CSC and AHA, the CCC-AF project is conducted by the Beijing Institute of Heart, Lung, and Blood Vessel Diseases from Beijing Anzhen Hospital. Being one of the participating hospitals in CCC-AF project, under the permission from host unit of Ethics Committee of Beijing Anzhen Hospital and our hospital, oral consent was obtained at the beginning of data collection instead of the formal one. Furthermore, data use, analysis and implementation of this study were agreed by host unit of Beijing Anzhen Hospital with signing formal consent.

### Definitions

Patients were diagnosed if AF or atrial flutter was presented on an electrocardiogram during admission or obtained from a hospital or physician chart. Every electrocardiographic record of AF was confirmed by at least two cardiologists. Identification of nonvalvular (NVAF) and valvular AF was based on CCS and ACC/AHA/Heart Rhythm Society guidelines [12]. Height and weight were measured using standardized protocol on admission. BMI was calculated as weight in kilograms divided by the square of height in meters (kg/m^2^). All patients were categorized into five groups according to BMI following the World Health Organization recommendations for Asians: underweight, < 18.5 kg/m^2^; normal range, 18.5–22.9 kg/m^2^; overweight, 23.0–24.9 kg/m^2^; obese class I, 25.0–29.9 kg/m^2^; and obese class II, ≥30.0 kg/m^2^ [13]. Hypertension was defined as systolic blood pressure ≥ 140 mmHg or diastolic blood pressure ≥ 90 mmHg upon the records of ambulatory blood pressure monitoring and blood pressure measurement in at least three successive days, or having a history of hypertension or receiving antihypertensive therapy. Diabetes mellitus was considered on the basis of history records or the intake of glucose-lowering drugs. Chronic heart failure (CHF), stroke, renal failure, rheumatic heart disease, chronic obstructive pulmonary disease, and cancer were all defined as self-reported medical history. CHADS_2_ and ChA_2_DS_2_-VASc scores are associated with an increased risk of stroke and death in patients with AF, and they also play predictive roles in clinical anticoagulant use. CHADS_2_ score (range: 0–6) includes congestive heart failure, hypertension, age ≥ 75 years, diabetes mellitus, and previous stroke/transient ischemic attack (double weight), which is the most commonly used parameter for stratifying stroke risk. CHA_2_DS_2_-VASc score (range: 0–9) adds items of vascular disease, age 65–74 years, (female) sex category based on CHADS2, and an alternative scoring of age with double weight to age ≥ 75 years [14].

### Statistical analysis

Basic information, medical procedure, and in-hospital outcomes of patients were described according to the BMI categories. Continuous variables were shown as mean ± standard deviation or median with 25th and 75th percentiles, whereas categorical variables were presented as count and percentages. Continuous or categorical variables were compared using unpaired *t*-test or one-way ANOVA test and χ^2^ test, respectively. The association between BMI and in-hospital outcomes was detected and further adjusted using binary logistic regression, which included age, sex, CHADS_2_ score, CHA_2_DS_2_-VAS score, heart failure history, stroke history, hypertension, and diabetes mellitus as adjusted variables. All *p* values were two-tailed, and *p* < 0.05 was considered statistically significant. Statistical analyses were performed using SPSS 23.0 (IBM) software.

## Results

### Baseline characteristics

Table 1 provides the baseline characteristics obtained for the study. Among the 15,867 patients with AF, the percentage of underweight, normal weight, overweight, obese class I, and obese class II individuals accounted for 5.2, 31.3, 23.4, 33.2, and 6.9%, respectively. Compared with those with normal weight, patients with higher BMI were younger and more exposed to smoke and drinking. Patients with higher BMI presented more history of hypertension and diabetes mellitus, whereas patients who were underweight previously suffered from CHF, renal failure, chronic obstructive pulmonary disease (COPD), and cancer. Furthermore, patients with low and high BMI presented higher CHADS_2_ scores (3–6) and CHA_2_DS_2_-VAS scores (5–9) compared with those with normal weight, respectively. Interestingly, overweight–obese AF patients had greater use of oral medications, including rhythm control drugs and anticoagulants, before admission than underweight–normal weight patients.

**Table 1:** Clinical characteristics of patients with AF on admission

Variables	Total *N* = 15,867	Underweight BMI < 18.5 (*n* = 830)	Normal 18.5 ≤ BMI < 23 (*n* = 4965)	Overweight 23 ≤ BMI < 25 (*n* = 3716)	Obese, class I 25 ≤ BMI < 30 (*n* = 5263)	Obese, class II BMI ≥ 30 (*n* = 1093)	*p* value
Age	68.09 ± 11.93	72.42 ± 12.69	69.46 ± 12.01	68.11 ± 11.65	66.62 ± 11.57	65.64 ± 11.90	< 0.001
Male	8860 (55.8)	352 (42.4)	2610 (52.6)	2158 (58.1)	3189 (60.6)	551 (50.4)	< 0.001
Current smoker	3421 (21.6)	147 (17.7)	990 (19.9)	764 (20.6)	1291 (24.5)	229 (21.0)	< 0.001
Current drinking	1808 (11.4)	60 (7.2)	476 (9.6)	412 (11.1)	722 (13.7)	138 (12.6)	< 0.001
**Comorbidities**							
Hypertension	8504 (53.6)	296 (35.7)	2306 (46.4)	1954 (52.6)	3205 (60.9)	743 (68.0)	< 0.001
Diabetes mellitus	2712 (17.1)	68 (8.2)	641 (12.9)	666 (17.9)	1064 (20.2)	273 (25.0)	< 0.001
CAD	3667 (23.1)	180 (21.7)	1073 (21.6)	909 (24.5)	1236 (23.5)	269 (24.6)	0.012
CHF	2194 (13.8)	171 (20.6)	747 (15.0)	500 (13.5)	642 (12.2)	134 (12.3)	< 0.001
Stroke	2024 (12.8)	113 (13.6)	628 (12.6)	489 (13.2)	658 (12.5)	136 (12.4)	0.820
Renal failure	396 (2.4)	39 (4.7)	129 (2.6)	81 (2.2)	130 (2.5)	17 (1.6)	< 0.001
Underwent dialysis	22 (5.5)	2 (5.1)	10 (7.8)	3 (3.7)	6 (4.6)	1 (5.9)	0.002
Renal transplant	7 (1.8)	0 (0)	3 (2.3)	0 (0)	4 (3.1)	0 (0)	0.001
Rheumatic heart disease	887 (5.6)	105 (12.7)	386 (7.8)	198 (5.3)	169 (3.2)	29 (2.7)	< 0.001
COPD	613 (3.9)	57 (6.9)	202 (4.1)	132 (3.6)	179 (3.4)	43 (3.9)	< 0.001
Cancer	260 (1.6)	27 (3.3)	105 (2.1)	61 (1.6)	55 (1.0)	12 (1.1)	< 0.001
First diagnosis of AF	1609 (10.1)	84 (10.1)	482 (9.7)	403 (10.8)	545 (10.4)	95 (8.7)	< 0.001
Non-valvular AF	13,792 (86.9)	610 (73.5)	4106 (82.7)	3265 (87.9)	4796 (91.1)	1015 (92.9)	< 0.001
**On admission value**							
HR at rest (bmp/min)	88.64 ± 27.68	93.45 ± 29.77	88.57 ± 27.90	88.05 ± 27.92	87.87 ± 26.85	91.03 ± 27.35	< 0.001
SBP (mmHg)	130.57 ± 20.45	123.69 ± 21.04	128.50 ± 20.57	130.50 ± 20.47	132.64 ± 19.85	135.45 ± 19.81	< 0.001
DBP (mmHg)	78.82 ± 13.33	74.11 ± 13.52	77.10 ± 13.12	78.69 ± 13.15	80.46 ± 13.21	82.74 ± 13.20	< 0.001
QTc (ms)	419.22 ± 64.13	416.15 ± 60.39	418.82 ± 65.16	417.48 ± 61.78	419.31 ± 62.92	428.15 ± 59.31	0.003
LVEDd (ms)	49.99 ± 8.18	48.26 ± 9.41	49.75 ± 8.71	49.82 ± 8.02	50.36 ± 7.74	51.00 ± 7.18	< 0.001
LAAP (mm)	42.73 ± 9.11	43.42 ± 11.75	42.39 ± 9.46	42.11 ± 9.21	43.02 ± 8.33	44.28 ± 8.46	< 0.001
LVEF							< 0.001
< 40%	1151 (7.3)	77 (9.3)	421 (8.5)	241 (6.5)	338 (6.4)	74 (6.8)	–
40–49%	1463 (9.2)	95 (11.4)	468 (9.4)	354 (9.5)	450 (8.6)	96 (8.8)	–
≥ 50%	13,253 (83.5)	658 (79.3)	4076 (82.1)	3121 (84.0)	4475 (85.0)	923 (84.4)	–
**CHADS**_**2**_**score**							0.001
0–1	9541 (60.1)	489 (58.9)	3058 (61.6)	2265 (61.0)	3126 (59.4)	603 (55.2)	–
2	3519 (22.2)	196 (23.6)	1088 (21.9)	773 (20.8)	1179 (22.4)	283 (25.9)	–
3–6	2782 (17.5)	141 (17.0)	809 (16.3)	670 (18.0)	955 (18.1)	207 (18.9)	–
**CHA**_**2**_**DS**_**2**_**-VAS score**							< 0.001
0–1	4646 (29.3)	184 (22.2)	1396 (28.1)	1101 (29.6)	1674 (31.8)	291 (26.6)	–
2–4	9082 (57.2)	519 (62.5)	2937 (59.2)	2107 (56.7)	2881 (54.7)	638 (58.4)	–
5–9	2114 (13.3)	123 (14.8)	622 (12.5)	500 (13.5)	705 (13.4)	164 (15.0)	–
**Medication history**							
Beta-blocker	4485 (28.3)	190 (22.9)	1289 (26.0)	1002 (27.0)	1633 (31.0)	371 (33.9)	< 0.001
CCB	2361 (14.9)	76 (9.2)	601 (12.1)	500 (13.5)	982 (18.7)	202 (18.5)	< 0.001
Antiarrhythmic agents	1404 (8.8)	35 (4.2)	378 (7.6)	329 (8.9)	540 (10.3)	122 (11.2)	< 0.001
Antiplatelet agents	1137 (7.2)	48 (5.8)	351 (7.1)	286 (7.7)	359 (6.8)	93 (8.5)	0.094
Warfarin	2097 (13.2)	118 (14.2)	653 (13.2)	509 (13.7)	668 (12.7)	149 (13.6)	0.127
Aspirin	3302 (20.8)	114 (13.7)	952 (19.2)	786 (21.2)	1185 (22.5)	265 (24.2)	< 0.001

Data are mean ± SD for normally distributed data and median and interquartile range for non-normally distributed data, or n (%)

*AF* indicates atrial fibrillation; *BMI* Body mass index; *CAD* Coronary artery disease; *CCB* Calcium channel blocker; *CHF* Chronic heart failure; *COPD* Chronic obstructive pulmonary disease; *DBP* Diastolic blood pressure; *HR* Heart rate; *LAAP* Left atrium diameter; *LVEDd* Left ventricular end-diastolic dimension; *SBP* systolic blood pressure; *LVEF* Left ventricular ejection fraction; *QTc* Corrected QT interval; CHADS_2_ score, congestive heart failure, hypertension, age 75 years of older, diabetes mellitus, prior stroke and TIA; CHA_2_DS_2_-VAS score, congestive heart failure, hypertension, age 75 years of older, diabetes mellitus, previous stroke/transient ischemic attack, vascular disease, age 65–74 years, female

### Management during hospitalization and discharge

**Fig. 1:**
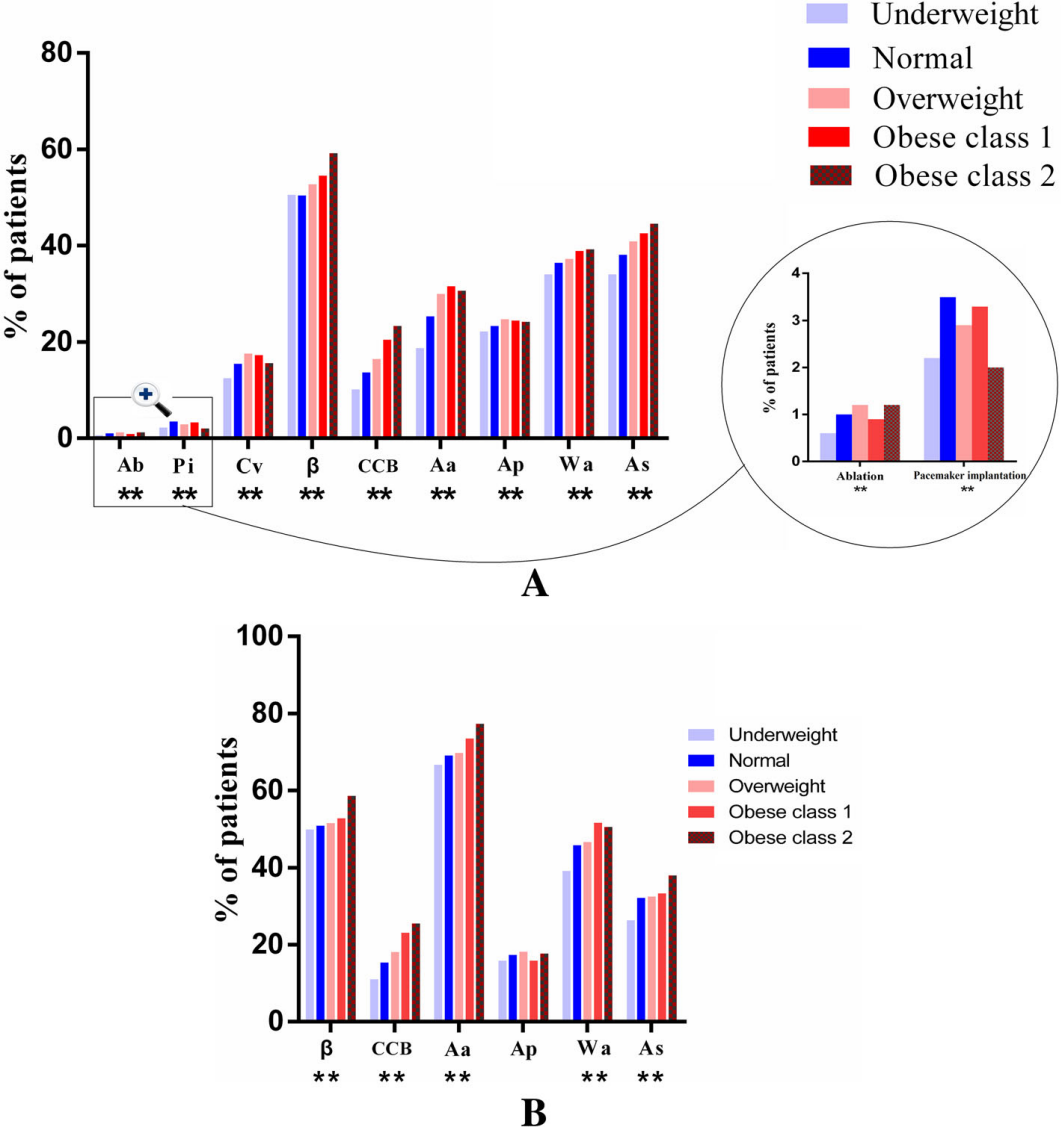
Medication and intervention therapies in hospitalization (**a**) and discharge (**b**) respectively according to BMI stratification. Ab indicates ablation; Pi, Pacemaker implantation; Cv, Cardioversion; β, beta-blocker; CCB, Calcium channel blocker; Aa, Antiarrhythmic agents; Ap, Antiplatelet agents; Wa, Warfarin; As, Aspirin. ** *p* < 0.01 among groups. † Treatment of calcium channel blocker included Dihydroarsenidine and non-Dihydroarsenidine. †† Treatment of cardioversion included drug and electrical cardioversion

Disparities in medical treatments and intervention among patients with AF stratified by BMI were observed (Fig. 1). Patients with high BMI obtained advantage in the use of oral medications. Except that of aspirin, the use of beta-blocker, calcium channel blocker (CCB), antiarrhythmic agents, and antiplatelet agents during hospitalization was higher in obese and overweight groups than in normal and underweight groups. Moreover, overweight patients more likely received cardioversion or ablation than normal-weight patients, who presented more pacemaker implantations than overweight patients. In obese patients, 32.9% received cardioversion therapy (including medical and electrical cardioversion), whereas 2.1% underwent catheter ablation, approximately 3 times more than underweight patients (all *p* < 0.001, Fig. 1a.) More details of specific value can be found from Supplementary material (Sup Table 1).

Among patients with AF, prescription rates at discharge of oral medications according to recommendations all increased compared with those before admission. However, compared with normal-weight patients, patients with low BMI categories were less likely to receive CCB, antiarrhythmic agents and aspirin at discharge (Fig. 1b).

### In-hospital outcomes

**Fig. 2:**
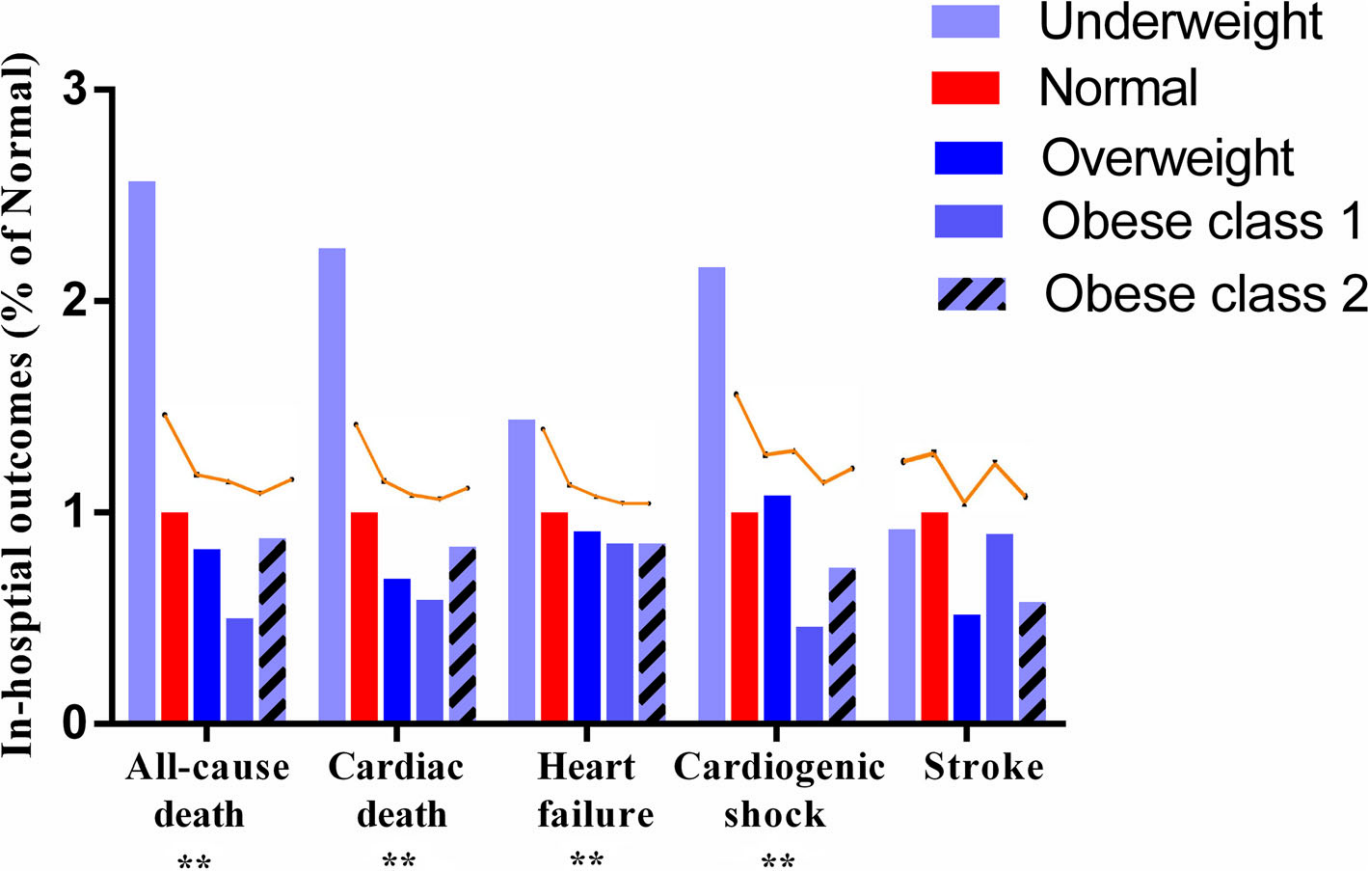
In-hospital outcomes among hospitalized patients with AF according to BMI stratification. † In-hospital outcomes in patients with normal weight were considered as references (the value was presented as 1 on Y-axis.). †† The orange curves above the columns presented the corresponding in-hospital outcomes as columns, and they shared the same data and statistical analysis, providing intuitive ratio change among groups. AF indicates atrial fibrillation. ** *p* < 0.01 among groups

**Fig. 3:**
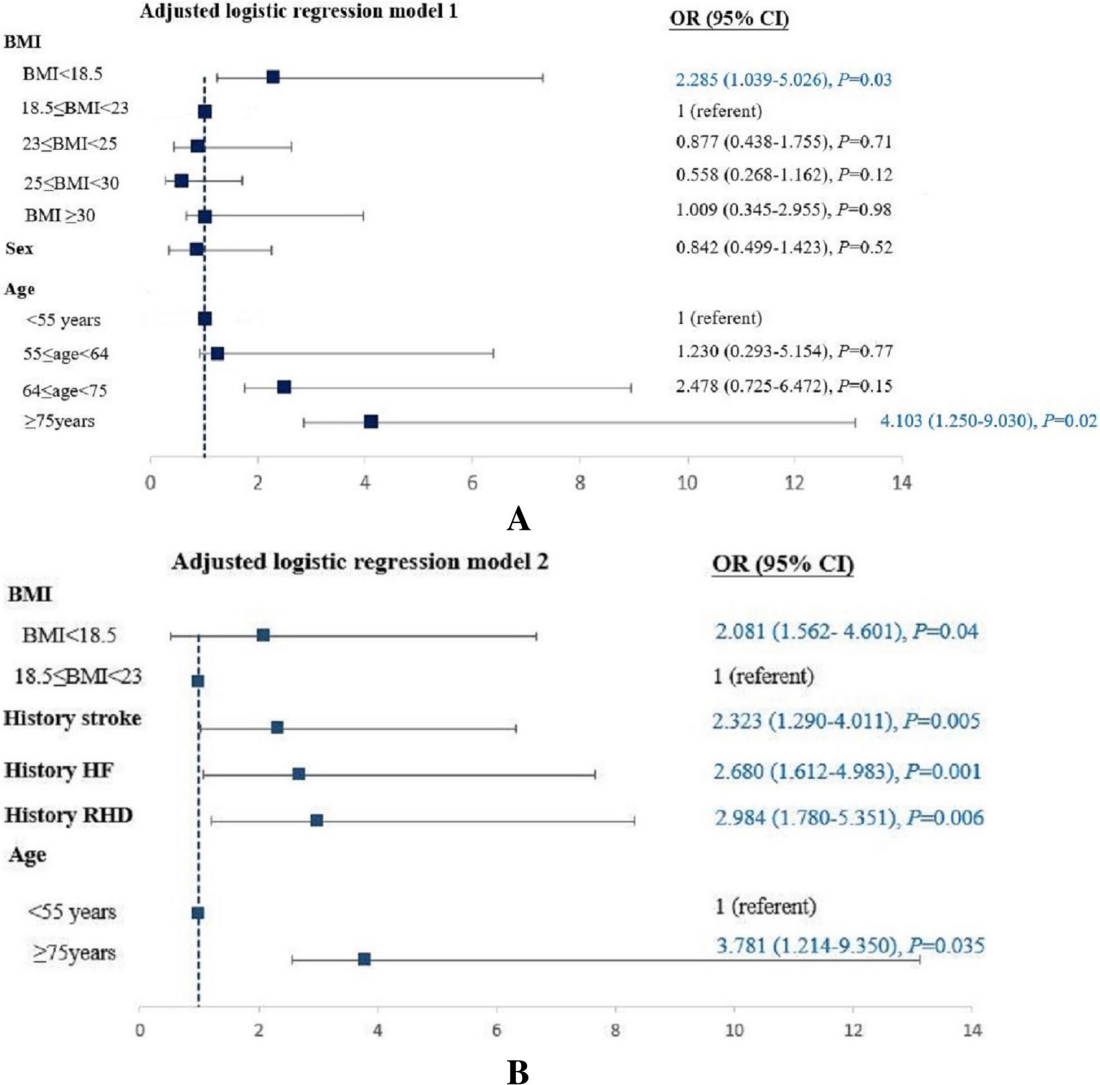
Regression analyses of association between BMI and all-cause mortality. With adjustment of age and sex only (**a**), and adjustment of sex, age, history of hypertension, diabetes mellitus, stroke, HF, renal failure, RHD, COPD, and cancer (**b**) in which the covariates were not included when lacking of statistical significance. BMI indicates body mass index; COPD, chronic obstructive pulmonary disease; HF, heart failure; OR, odds ratios; RHD, rheumatic heart disease

As illustrated in Fig. 2, worse in-hospital outcomes were observed in underweight patients than normal weight, overweight, and obese class I, including all-cause mortality (1.08% vs. 0.42, 0.35, and 0.21%, respectively, *p* = 0.003), cardiogenic shock (1.08% vs. 0.50, 0.54, and 0.23%, respectively, *p* = 0.006), and especially heart failure (23.97% vs. 16.60, 15.10, and 14.16%, respectively, *p* < 0.001). However, no statistical difference was observed in stroke (1.45% vs. 1.57, 1.16, and 1.44%, respectively, *p* = 0.343) among the groups. After adjustment for age and sex, underweight AF population showed higher risk of in-hospital mortality (adjusted odds ratio (OR), 2.29; 95% confidence interval (CI), 1.04–5.03; *p* = 0.03) than those who were overweight and obese class I (Fig. 3a). We further adjusted for hypertension, mellitus diabetes, stroke, heart failure, renal failure, rheumatic heart disease, COPD, and cancer, and BMI. Nevertheless, being underweight was still associated with higher incidence of in-hospital mortality (adjusted OR, 2.08; 95% CI, 1.56–4.60; *p* = 0.045) than being overweight and obese class I (Fig. 3b). Multivariable analyses indicated that AF patients with a history of heart failure (OR, 2.68; 95% CI, 1.61–4.98; *p* = 0.001), stroke (OR, 2.32; 95% CI, 1.29–4.01; *p* = 0.005), and rheumatic heart disease (OR, 2.98; 95% CI, 1.78–5.35); *p* = 0.006) and with age of ≥75 years (OR, 3.78; 95% CI, 1.21–9.35; *p* = 0.035) presented higher risk of in-hospital mortality.

As presented in Table 2, overweight patients with AF showed lower resting heart rate on discharge compared with underweight–normal patients. Interestingly, as the BMI increased, the length of stay during hospitalization reduced inversely. Although guidance on smoking, dietary patterns and weight management was provided to AF patients with slight bias to obese groups, a gap was still needed to increase changes in lifestyle and bad habits.

**Table 2:** Differences of clinical index and advisement among patients with AF on discharge

Variables	Total *N* = 15,867	Underweight BMI < 18.5 (*n* = 830)	Normal 18.5 ≤ BMI < 23 (*n* = 4965)	Overweight 23 ≤ BMI < 25 (*n* = 3716)	Obese, class I 25 ≤ BMI < 30 (*n* = 5263)	Obese, class II BMI ≥ 30 (*n* = 1093)	*p* value
HR at rest (bmp/min)	76.24 ± 17.50	77.96 ± 20.04	76.69 ± 17.89	75.00 ± 16.47	76.26 ± 17.42	77.27 ± 17.46	0.002
Length of stay, days	9.17 ± 8.77	9.83 ± 6.96	9.27 ± 7.36	9.28 ± 12.21	8.98 ± 7.73	8.76 ± 6.13	0.031
Guidance on quitting smoking	3120 (19.7)	142 (17.1)	921 (18.5)	705 (19.0)	1147 (21.8)	205 (18.8)	< 0.001
Guidance on dietary patterns	14,157 (89.2)	741 (89.3)	4423 (89.1)	3304 (88.9)	4712 (89.5)	977 (89.4)	0.007
Weight management	11,032 (69.5)	497 (59.9)	3306 (66.6)	2583 (69.5)	3801 (72.2)	845 (77.3)	< 0.001

Data are mean ± SD for normally distributed data and median and interquartile range for non-normally distributed data, or n (%)

*AF* indicates atrial fibrillation; *BMI* Body mass index; *HR* Heart rate

## Discussion

In this large multicenter-based registry for hospitalized AF patients in China, patients who were underweight presented more history of CHF, renal failure, and cancer and higher scores of CHADS2 and CHA2DS2-VAS than obese participants. Furthermore, underweight patients (BMI < 18.5) were less likely to receive oral medications and cardiac intervention therapies than normal- weight and overweight individuals. The underweight group exhibited higher crude in-hospital death, heart failure, and cardiogenic shock rate than normal-weight or obese patients. After multivariable adjustment, underweight but not overweight patients with AF in China showed association with increased risk of in-hospital mortality, and low BMI prefer to co-morbidities and advanced age may be one of the possible explanations in our study.

Obesity features strong relationships not only with increased risk of NOAF [15] but also the subsequent progression of AF compared with normal weight [5, 16]. However, we observed an increased risk of in-hospital mortality among underweight patients with AF rather than obese ones in China. Underweight phenotype is suggested to be associated with the presence of cardiovascular disease (CVD) in ICD I00-I99 or a history of myocardial infarction [6, 17]. The worsened progression of disease may be caused by the generally low prescription rates during hospitalization and subsequent maintenance therapies after discharge. In this hospital-based study, the prescription rates, including those for aspirin, beta-blockers, antiplatelet agents, and CCB, from both hospitalization and discharge were lower in underweight participants than in normal- weight or obese groups. Similar to several studies, being underweight was associated with high frequency of chronic lung disease, malignancy, and perhaps thyroid disease [6, 17]. Comprehensive factors, including physical conditions, dietary status, and physical endurance of underweight patients may affect medical and intervention therapies from physicians.

Obesity is thought to be one of the risk factors responsible for incidence of CVD [18]. Interestingly, in patients with heart failure, CAD and AF, obesity is positively associated with better outcomes [19–21]. In this study, obese class I (25 ≤ BMI < 30) was negatively associated with in-hospital mortality by using unadjusted binary regression model. However, it showed no relationship after adjustment for age, sex, and other clinical complications. Such finding is especially relevant as age is one of the major predictors of all-cause mortality among patients with AF [22]. From this aspect, the phenomenon is referred to as “obesity paradox” and the obesity mortality paradox in AF may be a true clinical case [23]. Several potential reasons are found for the paradoxical results of obesity in AF, including undesirable factors, such as the effect of comorbidities (hypertension, diabetes mellitus, obstructive sleep apnea syndrome etc.), pericardial fat deposits and highly metabolic active tissue, the influence of prevalent cardiac dysfunctions (ventricular adaptation, greater left atrial size etc.), and an increased activity of the sympathetic nervous system associated with obesity. The positive relationship between obesity and outcomes of AF was due to the ability of obesity-linked lipoproteins in blood to scavenge bacterial lipopolysaccharide, diminishing the effect of inflammatory process activity and the reducing effect of obesity on the blood concentration of atrial natriuretic peptide [24]. In this study, overweight and obese patients were younger, experienced more hypertension, and featured slightly higher left atrium diameter value and heart rate than those with underweight and normal weight. This result implies that underweight–normal-weight patients may exhibit lower metabolic reserves to counterbalance the increased catabolic stress of AF than obese patients.

The associations between underweight status and all-cause mortality may be explained as follows. Inflammation is more active and exists longer in obese patients than that in normal-weight or underweight population and results from chronic inflammatory conditions, including mediator production and interactions among cytokines and cells [25, 26]. To date, limited research identified these complicated elements or the abovementioned changes in the underweight population. Several studies on cancer reported that underweight always follow poor nutritional status, which is associated with increased toxicity and decreased response to anti-cancer therapy [27, 28]. By contrast, overweight and obese patients consistently maintain larger nutritional stores and have been shown to present significantly lower rates of treatment-related toxicity than underweight patients. However, underweight patients may include both undernourished and healthy individuals with a lean body type. Serum albumin, prognostic nutritional index (PNI), and hemoglobin levels were significantly lower in the underweight population than in others from a present study [29]. Concordant with other research [30], our study showed that underweight AF patients presented lower prescription rate and intervention therapies during hospitalization than those with normal weight or obesity, indicating the same trends as medicine usage on discharge. In general, compared with underweight AF patients, those belonging to high BMI categories benefit from younger age; fewer serious comorbidities, such as renal dysfunction or cancer; greater use of anticoagulants; and rhythm control therapies. However, increasing adipose tissues may become a relatively beneficial factor to obese patients. Adiponectin, which is produced by adipose tissue, exerts anti-inflammatory and cardioprotective effects, including in myocardial infarction and CAD [31]. Underweight patients present higher adiponectin levels than those with normal weight [32]. High adiponectin levels have been found to be associated with an increased risk of AF [33]. Regardless of adjustment, our study suggested that being underweight retained the negative correlation with all-cause mortality in patients with AF during hospitalization.

### Limitations

Despite the strengths listed above, several limitations deserve to be mentioned. First, although BMI is conventionally thought to be associated with outcomes of AF, differences in body fat distribution are nowadays considered more important than calculated BMI alone. Waist circumference and waist-to-hip circumference ratio or pericardial fat value were excluded from this registry research, but they could have provided additional information. Second, AF was determined by registration data, and early recognition of AF and quantification of AF burden are aggravated by the often-silent nature of the rhythm disturbance (e.g., asymptomatic AF). Therefore, we cannot ensure that any asymptomatic or unrecognized AF existed before the patients were enrolled into the study. We could not analyze the subtypes of AF (including initial, paroxysmal, persistent, and permanent AF) separately. The subtypes and duration of disease could influence metabolic derangements, left ventricular hypertrophy, and subsequent progressive atrial dilatation. These conditions may also contribute to BMI-related outcomes and may become further prospective research interests. Third, as one of the highly complex arrhythmias, AF must be managed with a multidisciplinary integrated care approach, including primary care physicians, dieticians, endocrine specialists, sleep physicians, and exercise physiologists, to improve recommended therapies and cardiorespiratory fitness in underweight, overweight and obese subjects. Retrospective studies failed to analyze significant differences in weight change during follow-up on the basis of BMI. Studies referring to the association between weight management following dieticians and long-term outcomes among hospital-based AF patients are needed in the future. Fourth, the underweight patients with AF suffered from more comorbidities and advanced age than any other BMI-stratified patients. The high rate of comorbidities and age in underweight patients with AF may explain their high in-hospital mortality in our study. Therefore, further studies are required for specific enrolled patients with AF stratified by BMI. Finally, our cohort predominantly comprised Chinese patients. Thus, our findings may not apply to non-Asian individuals. Despite these limitations, our study provides important real-world data on the relationship between BMI and clinical outcomes in AF patients.

## Conclusions

In this observational study, BMI and all-cause mortality in AF patients showed an inverse relationship. Underweight AF patients. Presented higher crude in-hospital death, heart failure, and cardiogenic shock rate than normal-weight or obese AF individuals. After multivariable adjustment, the possible causes of low BMI may associate with other comorbidities and advanced age. Underweight BMI in Chinese patients but not overweight status was associated with increased risk of in-hospital mortality.

## Supplementary information

**Additional file 1:Table S1.** BMI-based differences in medication strategies before, during hospitalization and discharge.

## Data Availability

The data that support the findings of this study are available from Beijing Anzhen Hospital, Capital Medical University but restrictions apply to the availability of these data, which were used under license for the current study, and so are not publicly available. Data are however available from the authors upon reasonable request and with permission of Beijing Anzhen Hospital, Capital Medical University.

## Abbreviations

AF: Atrial fibrillation
BMI: Body mass index
CAD: Coronary artery disease
CHF: Chronic heart failure
CCB: Calcium channel blocker
CCC-AF project: Care for cardiovascular disease in China- atrial fibrillation project
CI: Confidence interval
NOAF: New-onset atrial fibrillation
NVAF: Nonvalvular atrial fibrillation
OR: Odds ratio
PNI: Prognostic nutritional index
ROS: Reactive oxygen species
