# Supplementary material for: BMI differences among in-hospital management and outcomes in patients with atrial fibrillation: findings from the Care for Cardiovascular Disease project in China
Source: BMC Cardiovasc Disord. 2020 Jun 5;20:270. doi: 10.1186/s12872-020-01544-8 (PMC7275422; doi:10.1186/s12872-020-01544-8)
Supplement: Supplementary file 1 — Additional file 1:Table S1. BMI-based differences in medication strategies before, during hospitalization and discharge. [file 12872_2020_1544_MOESM1_ESM.docx]

**Sup. Table 1** BMI-based differences in medication strategies before, during hospitalization and discharge

| **Variables** | Total  N=15867 | Underweight  BMI<18.5  (n=830) | Normal  18.5≤BMI<23  (n=4965) | Overweight  23≤BMI<25  (n=3716) | Obese, class Ⅰ  25≤BMI<30  (n=5263) | Obese, class Ⅱ  BMI≥30  (n=1093) | p value |
| --- | --- | --- | --- | --- | --- | --- | --- |
| **Before admission**, n (%) |  |  |  |  |  |  |  |
| Beta-blocker | 4485 (28.3) | 190 (22.9) | 1289 (26.0) | 1002 (27.0) | 1633 (31.0) | 371 (33.9) | 0.000 |
| CCB | 2361 (14.9) | 76 (9.2) | 601 (12.1) | 500 (13.5) | 982 (18.7) | 202 (18.5) | 0.000 |
| Antiarrhythmic agents | 1404 (8.8) | 35 (4.2) | 378 (7.6) | 329 (8.9) | 540 (10.3) | 122 (11.2) | 0.000 |
| Antiplatelet agents | 1137 (7.2) | 48 (5.8) | 351 (7.1) | 286 (7.7) | 359 (6.8) | 93 (8.5) | 0.094 |
| Warfarin | 2097 (13.2) | 118 (14.2) | 653 (13.2) | 509 (13.7) | 668 (12.7) | 149 (13.6) | 0.127 |
| Aspirin | 3302 (20.8) | 114 (13.7) | 952 (19.2) | 786 (21.2) | 1185 (22.5) | 265 (24.2) | 0.000 |
| **During hospitalization**, n (%) |  |  |  |  |  |  |  |
| Ablation# | 162 (1.02) | 5 (0.6) | 51 (1.0) | 46 (1.2) | 47 (0.9) | 13 (1.2) | 0.000 |
| Cardioversion## | 2615 (16.5) | 104 (12.5) | 770 (15.5) | 657 (17.6) | 913 (17.3) | 171 (15.6) | 0.000 |
| Pacemaker implantation | 494 (3.1) | 18 (2.2) | 174 (3.5) | 108 (2.9) | 172 (3.3) | 22 (2.0) | 0.000 |
| Antiarrhythmic agents | 4530 (28.5) | 156 (18.8) | 1260 (25.4) | 1116 (30.0) | 1662 (31.6) | 336 (30.7) | 0.000 |
| Antiplatelet agents | 3824 (24.1) | 184 (22.2) | 1164 (23.4) | 922 (24.8) | 1290 (24.5) | 264 (24.2) | 0.000 |
| Aspirin | 6420 (40.5) | 282 (34.0) | 1891 (38.1) | 1520 (40.9) | 2240 (42.6) | 487 (44.6) | 0.000 |
| Warfarin | 5943 (37.5) | 282 (34.0) | 1806 (36.4) | 1382 (37.2) | 2044 (38.9) | 429 (39.2) | 0.000 |
| CCB | 2715 (17.1) | 85 (10.2) | 681 (13.7) | 613 (16.5) | 1080 (20.5) | 256 (23.4) | 0.000 |
| Beta-blocker | 8402 (53.0) | 420 (50.6) | 2501 (50.4) | 1961 (52.8) | 2873 (54.6) | 647 (59.2) | 0.000 |
| **Discharge**, n (%) |  |  |  |  |  |  |  |
| Aspirin | 5196 (32.7) | 219 (26.4) | 1597 (32.2) | 1206 (32.5) | 1759 (33.4) | 415 (38.0) | 0.000 |
| Warfarin | 7601 (47.9) | 325 (39.2) | 2275 (45.8) | 1732 (46.6) | 2717 (51.6) | 552 (50.5) | 0.000 |
| Antiplatelet agents | 2705 (17.0) | 132 (15.9) | 865 (17.4) | 677 (18.2) | 838 (15.9) | 193 (17.7) | 0.183 |
| Beta-blocker | 8223 (51.8) | 414 (49.9) | 2528 (50.9) | 1913 (51.5) | 2781 (52.8) | 641 (58.6) | 0.000 |
| CCB | 3020 (19.0) | 91 (11.0) | 763 (15.4) | 671 (18.1) | 1216 (23.1) | 279 (25.5) | 0.000 |
| Antiarrhythmic agents | 11291(71.2) | 553 (66.7) | 3432 (69.1) | 2594 (69.8) | 3866 (73.5) | 846 (77.4) | 0.000 |
| Smoking cessation counseling† | 3114 (19.6) | 141 (17.0) | 919 (18.5) | 704 (18.9) | 1146 (21.8) | 204 (18.7) | 0.000 |

# Ablation includes radiofrequency ablation and Cryoablation. ## Cardioversion includes medical and electrical cardioversion. † Smoking cessation counseling includes stop-smoking prescription medicines, dissemination of publicity materials, oral advice and stop-smoking program planning. CCB indicates Calcium channel blocker.
